# Immunogenicity and Protective Efficacy of a Multi-Antigen *Mycobacterium tuberculosis* Subunit Vaccine in Mice

**DOI:** 10.3390/vaccines12090997

**Published:** 2024-08-30

**Authors:** Annuurun Nisa, Rachel Pinto, Warwick J. Britton, James A. Triccas, Claudio Counoupas

**Affiliations:** 1 Faculty of Medicine and Health, The University of Sydney Infectious Diseases Institute (Sydney ID), Camperdown, NSW 2050, Australia; anis9013@alumni.sydney.edu.au (A.N.); rachel.pinto@sydney.edu.au (R.P.); 2 School of Medical Sciences, Faculty of Medicine and Health, The University of Sydney, Camperdown, NSW 2050, Australia; 3 Centre for Infection and Immunity, Centenary Institute, The University of Sydney, Camperdown, NSW 2050, Australia; w.britton@centenary.org.au; 4 Department of Clinical Immunology, Royal Prince Alfred Hospital, Camperdown, NSW 2050, Australia

**Keywords:** tuberculosis, vaccines, T cells, immune response, antigen

## Abstract

There is an urgent need for an effective TB vaccine capable of controlling both acute and chronic *Mycobacterium tuberculosis* infection in populations with diverse genetic backgrounds. In this study, we characterised the immunogenicity and protective efficacy of a novel protein-in-adjuvant subunit vaccine. The protein component is a fusion protein of three different *M. tuberculosis* antigens, which we termed CysVac5: CysD, a major component of the *M. tuberculosis* sulfate activation pathway that is highly expressed during the chronic stage of *M. tuberculosis* infection, is fused with two major secreted mycobacterial antigens, Ag85B and MPT83. Vaccination of C57BL/6 mice with CysVac5, formulated in a monophosphoryl lipid A (MPLA) and dimethyldioctadecylammonium (DDA) adjuvant combination, resulted in the potent generation of polyfunctional CD4^+^ T cells secreting multiple cytokines, including IFN-γ, IL-2, TNF and IL-17, against each of the three components of the fusion protein. Furthermore, vaccination with CysVac5-MPLA/DDA conferred significant protection against infection in mouse lungs, which was greater than that afforded by BCG at extended time points post-challenge. The generation of antigen-specific and protective immunity was also observed in CysVac5 vaccinated BALB/c mice, indicating the vaccine could display efficacy across multiple genetic backgrounds. These results indicate that the CysVac5 vaccine has broad immunogenicity, is effective in controlling both acute and chronic phases of *M. tuberculosis* infection in mice, and warrants further investigation to assess its potential to control pulmonary TB.

## 1. Introduction

Tuberculosis (TB) is a leading global cause of death from a single infectious agent, causing over 1.3 million deaths in 2022 [1]. The coronavirus disease 2019 (COVID-19) pandemic has negatively impacted TB diagnosis and care, resulting in a reversal of the progress made up to 2019 in combating TB [2]. It is estimated that a quarter of the world’s population is latently infected with *Mycobacterium tuberculosis*, allowing the bacteria to survive and enter a quiescent phase that can reactivate later in life. *Mycobacterium bovis* Bacillus Calmette–Guérin (BCG) is the only licensed TB vaccine available for humans. Despite being able to provide good protection for newborns and children against disseminated forms of TB, BCG displays a high variation in its degrees of protection against pulmonary TB infection in adolescents and adults (ranging from 0–80%), the population that bears the highest burden of the disease [3]. Therefore, improved vaccines that offer efficient and consistent protection against this disease are urgently needed.

Several novel vaccine candidates against TB are currently in preclinical and clinical trials, including recombinant BCG (rBCG) strains, live attenuated *M. tuberculosis* strains, mycobacterial extracts, recombinant viral-vectored platforms, mRNA, and protein–adjuvant combinations (reviewed in [4]). Subunit vaccines have the advantage of being safer compared to live vaccines, especially in immunocompromised individuals, and may be given as multiple-dose regimens. Many pre-clinical and clinical studies have shown the ability of protein-based subunit vaccines to induce long-lasting T-cell responses; therefore, they are able to provide long-term protection against *M. tuberculosis* infection [5,6,7,8]. In a landmark study, the M72/AS01E candidate exhibited 49.7% efficacy in preventing active pulmonary tuberculosis disease among adults with *M. tuberculosis* infection [9]. This protective signature was characterised by a sustained elevation of M72-specific antibodies and CD4^+^ T cells.

Central to the development of successful TB subunit vaccines is the careful selection of antigens that can elicit robust and protective immune responses in humans. This selection process is influenced by several key factors, including the immunogenicity of the antigens in the human population and their expression by the bacteria at different stages of infection. During infection, mycobacteria encompass a diverse range of bacterial populations, existing in distinct stages of metabolic activity and exhibiting varied antigen expression profiles. This includes both actively replicating bacteria and dormant forms, with the ability to switch between these metabolic states. Many vaccine studies include different antigens in their study, often in the form of fusion proteins [10,11]. Including multiple antigens from *M. tuberculosis* as candidates for TB subunit vaccines can contribute to broadening immunogenicity and enhancing the protective efficacy across populations with different HLA compositions. Previous studies have identified a protein family comprising the *M. tuberculosis* sulphate-assimilation pathway (SAP), whose members are highly upregulated during conditions of intracellular stress [12]. Protein components of the SAP pathway are immunogenic and recognised by both murine and human T cells during the course of *M. tuberculosis* infection, and when tested as vaccine antigens, these proteins were protective in murine challenge models [13,14]. The fusion protein vaccine CysVac2 (CysD fused with the mycobacterial antigen Ag85B), when delivered with MPLA/DDA, was highly immunogenic, inducing both CysD- and Ag85B-specific polyfunctional Th1- and Th17-type CD4^+^ T-cell responses. CysVac2 was also highly protective in both pre- and post-exposure models of *M. tuberculosis* infection in mice at the chronic stage of infection, and when given as a BCG booster, CysVac2 was able to improve the protection afforded by prior BCG vaccination [14,15]. When overexpressed by recombinant BCG, it also enhanced protection at the chronic stages of *M. tuberculosis* infection [16].

MPT83 (Rv2873) is a surface-expressing lipoglycoprotein of *M. tuberculosis* and an analogue to MPB83 in *M. bovis*. MPT83 was upregulated after *M. tuberculosis* infection of mice and functions as a highly immunogenic antigen [17]. In addition, MPT83 was recognised by the immune system of patients with active TB. Subcutaneous immunisation with MPT83 protein vaccines induced significant protection against aerosol *M. tuberculosis* challenge in mice [18], and the protein has successfully been used as a component of protective TB fusion antigens [11]. MPT83 is a *M. tuberculosis* proapoptotic protein, which could contribute to its ability to stimulate strong immunity [19]. In this study, we investigated the effect of modifying the CysVac2 fusion protein by the addition of the MPT83 antigen, resulting in the CysVac5 vaccine. Here, we examined the capability of this fusion protein to provide broad immune responses and protection against *M. tuberculosis* challenge in two different strains of mice.

## 2. Materials and Methods

Bacterial strains and growth conditions. *E. coli* DH5α and BL21 (DE3) were grown in Luria–Bertani (LB) broth or agar (Difco BD, Franklin Lakes, NJ, USA). *M. bovis* BCG Pasteur (ATCC35734) and *M. tuberculosis* H37Rv (ATCC27294; BEI Resources, NIAID, NIH, NR-13648) were cultured in Middlebrook 7H9 (Difco BD) broth supplemented with albumin-dextrose-catalase (ADC; 10% *v*/*v*), Tween-80 (0.05% *v*/*v*) and glycerol (0.2% *v*/*v*). To enumerate the bacterial numbers, cultures were plated onto Middlebrook 7H11 (Difco BD) agar with oleic-acid-albumin-dextrose catalase (OADC; 10% *v*/*v*) and glycerol (0.5% *v*/*v*). All cultures were grown at 37 °C with or without shaking. The antibiotic kanamycin was added to the media when required at 50 μg/mL.

Recombinant CysVac5 expression and purification. The recombinant prokaryotic expression plasmid pET28a-CysVac5 was constructed by fusing the gene encoding the secreted antigen FbpB (Ag85B; Rv1886C) with downstream insertion of *cysD* (Rv1285) and *mpt83* (Rv2873) genes from *M. tuberculosis* genomic DNA. Plasmids were transformed into *E. coli* BL21 (DE3), and selected transformants were grown until the mid-log phase (OD_600_ of 0.5–0.6). CysVac5 expression was induced by 0.5 M of isopropyl β-D-1-thiogalactopyranoside (IPTG) shaking overnight at 37 °C. The bacterial culture was pelleted by centrifugation, resuspended in lysis buffer (50 mM KPO_4_, 4 mM KCl, 5 mM b-ME, lysozyme 0.1 mg/mL, pH 8) and sonicated with a probe sonicator (Branson, Milford, CT, USA). The soluble and insoluble fractions were separated at 12,000 rpm for 1 h at 4 °C (Eppendorf, Hamburg, Germany). The insoluble fraction was resuspended overnight at 4 °C in urea buffer (8 M urea, pH 8) and then passed through a His-tag affinity chromatography column (Clontech, Mountain View, CA, USA). After several washes with urea buffer with increasing concentrations of imidazole, the His-tagged protein was eluted from the column in urea buffer containing 200 mM imidazole and then dialysed overnight in a refolding buffer (25 mM HEPES, 0.1 M KCl, 1.5 mM DTT, 5% glycerol, pH 7.5). The protein concentration was measured by the Bradford assay, and the protein expression was confirmed by sodium dodecyl sulphate-polyacrylamide gel electrophoresis (SDS-PAGE) and Western blot, as described previously [14].

Preparation of live mycobacterial and MPLA/DDA liposomal adjuvanted vaccine. *M. bovis* BCG Pasteur strain was grown in 7H9 broth media with appropriate antibiotics to the mid-log phase (OD_600_ of 0.5–0.6). Bacterial cultures were then transferred into a 50 mL falcon tube and pelleted by centrifugation (3500 rpm, 10 min, room temperature). The supernatant was removed, and the bacterial cell pellet was resuspended in 1 mL of sterile PBS. Prior to infection, the cells were briefly sonicated (Branson) to disperse aggregated mycobacteria. Dimethyl dioctadecyl ammoniumbromide (DDA; 5 mg/mL; Sigma-Aldrich, St. Louis, MO, USA) was prepared in sterile water and heated to 80 °C with regular stirring for 20 min, whereas monophosphoryl lipid A (MPLA; InvivoGen, San Diego, CA, USA) was resuspended in 1 mL of DMSO and vortexed to allow complete solubilisation. The formulation of MPLA/DDA adjuvant was prepared in a ratio of 1:10 (*w*/*w*; 25 µg of MPLA with 250 µg of DDA).

Mice and immunisation procedures. Female C57BL/6 or BALB/c mice (6–8 weeks old) were obtained from the Animal Resources Centre (Perth, Australia). Mice were maintained in a specific pathogen-free condition at the Centenary Institute animal facility. All murine experiments were performed in strict accordance with the approvals granted by the University of Sydney Animal Ethics Committee (K75/9-2012/3/5846). Experiments were not blinded, and animals were randomly assigned to experimental groups. For subcutaneous (s.c.) administration, mice were anaesthetised with gaseous isofluorane (4%) and injected at the base of the tail with 3 μg of recombinant protein (CysVac2 or CysVac5) formulated in MPLA/DDA (25 μg/250 μg per dose, respectively; 200 μL total volume). Mice received three vaccinations 2 weeks apart and proceeded to the immunogenicity study at 2 and 6 weeks post-last vaccination, or *M. tuberculosis* challenge. Mice receiving BCG were vaccinated s.c. once with 5 × 10^5^ CFU 10 weeks prior to *M. tuberculosis* challenge.

*M. tuberculosis* infection. Six weeks following the final vaccination, mice were challenged with *M. tuberculosis* H37Rv in an inhalation exposure system using a Middlebrook airborne infection apparatus (Glas-Col, Terre Haute, IN, USA) with an infective dose of approximately 100 CFU. At 4 and 20 weeks after challenge, serial dilutions of lungs and spleen homogenates were plated on supplemented Middlebrook 711 agar (Difco BD) to enumerate the bacterial loads.

Organ collection and processing. Mice were held in a restraint, and a scalpel blade was used to puncture the tail lateral vein. Approximately 200 μL of blood was collected from mice in PBS-heparin (20 U/mL; Sigma-Aldrich), and the suspension was stratified on Histopaque 1083 (Sigma-Aldrich) and centrifuged. The peripheral blood mononuclear cells (PBMCs) were collected and washed twice with RPMI medium (Thermo Fisher Scientific, Waltham, MA, USA). Whole lungs from euthanised mice were perfused post-mortem with cold PBS-heparin. For isolation of lung leukocytes, lung tissue in complete RPMI medium, consisting of L-glutamine and 25 mM Hepes (Invitrogen, Waltham, CA, USA), FCS (10% *v*/*v*), 2-mercaptoethanol (50 μM; Sigma-Aldrich) and PenStrep (100 U/mL; Invitrogen), was digested with collagenase IV (50 U/mL; Sigma-Aldrich) and DNAse I (13 μg/mL; Sigma-Aldrich) at 37 °C for 45 min prior to homogenisation (using a GentleMACS Dissociator, Miltenyi Biotec Australia, Macquarie Park, Australia) and multiple filtration steps. Spleens were homogenised by passing through a 40 μm cell strainer (BD Biosciences, San Jose, CA, USA) in RPMI medium and then pelleted by centrifugation. Erythrocytes were removed using ACK lysis buffer, and single-cell suspensions were prepared and counted by Trypan Blue (0.04%) exclusion followed by dilution to the desired concentration.

Intracellular cytokine staining and flow cytometry. Antigen-specific cytokine production was measured by intracellular immunostaining (ICS) and flow cytometry. The antigens utilised were single components of the recombinant protein vaccine (Ag85B, CysD and MPT83), as well as fusion protein CysVac2 and CysVac5. Up to 5 × 10^6^ cells/mL of leukocytes were stimulated for 3–4 h (37 °C, 5% CO_2_) with appropriate antigens (10 μg/mL). Cells were further incubated for 16 h with Brefeldin A (10 μg/mL; Sigma-Aldrich) to allow cytokine accumulation prior to immunostaining. Cell suspensions were incubated with 1.25 μg/mL anti-mouse CD16/CD32 (2.4G2; BD Biosciences) in FACS wash buffer (PBS with 2% FCS) to block Fc receptors, then washed and incubated for 30 min with antibody mix to label surface markers and Fixable Blue Dead Cell stain (Thermo Fisher Scientific) to differentiate dead cells. Cells were permeabilised using BD Cytofix/Cytoperm kit (BD Biosciences). Intracellular staining was performed using appropriate antibodies, as shown in Supplementary Table S1. Immunostained cells or beads were fixed in 10% buffered formalin prior to the data acquisition using an LSR-Fortessa analyser (BD Biosciences). Acquired data was analysed using FlowJo analysis software version 10.10 (Treestar Inc., Ashland, OR, USA). A Boolean gate combination was used to calculate the frequency of single- or multiple-cytokine-positive cell subsets (the gating strategy is described in Supplementary Figure S1).

Murine IFN-γ T-cell responses. Membranes of a 96-well ELISPOT filter plate (Millipore, Darmstadt, Germany) were coated (overnight, 4 °C) with anti-IFN-γ antibody (10 μg/mL) in PBS, then washed and blocked with complete RPMI medium (Thermo Fisher Scientific) for 2 h at 37 °C. Splenocyte suspensions were plated at 2 × 10^5^ cells per well. Purified vaccine peptides or proteins (10 μg/mL), Concanavalin A (3 μg/mL) or medium alone were added and incubated for 20 h at 37 °C in 5% CO_2_. Plates were washed 6 times with PBST (0.1% Tween 20), and captured IFN-γ was detected with biotinylated anti-IFN-γ antibody (XMG1.2-biotin; 5 μg/mL in PBS/0.5% BSA, incubated overnight, 4 °C). Plates were washed, followed by the addition of avidin alkaline phosphatase (Sigma-Aldrich; 1:1000 *v*/*v* in PBS/0.5% BSA, 45 min, RT). The presence of IFN-γ-producing cells was visualised by the addition of alkaline phosphatase substrate solution (in NPP buffer; 0.01% Substrate A, 0.01% Substrate B; AP conjugate substrate kit, Bio-Rad, Hercules, CA, USA). Developed spots were then counted using an automated ELISPOT reader (AID EliSpot Reader software v 6.0; AID GmbH, Strassberg, Germany) and reported as frequency per million cells.

Histopathology. For histopathological analysis, the middle right lobe of each infected murine lung was harvested and then perfused with a 10% neutral buffered formalin solution. Tissue samples were embedded in paraffin, and 5 μm thick tissue sections were collected and stained with hematoxylin and eosin (H&E). Tissue slides were then visualised and observed using a Leica DM6000B microscope (Leica Microsystem©, North Ryde, NSW, Australia) with a magnification of 10× and acquired as mosaics. The average relative area of the granulomatous tissue in the lung was quantified using Adobe Photoshop CC 2015.15 [15] (Adobe Inc., San Jose, CA, USA). The percentages of granulomatous tissue were calculated as a percent of the total lung section area, as described previously by Nusbaum et al., 2016 [20].

Statistical analysis. Statistical analysis was performed using GraphPad Prism 10.3.1 software (GraphPad Software, La Jolla, CA, USA). The significance of differences between experimental groups was evaluated by one- or two-way analysis of variance (ANOVA), with a pairwise comparison of multi-grouped data sets achieved using Tukey’s multiple comparisons test and was considered significant when the *p* values were ≤0.05.

## 3. Results

### 3.1. CysVac5-MPLA/DDA Vaccination Induces High Frequency of Circulating Multifunctional CD4^+^ T Cells

**Figure 1:**
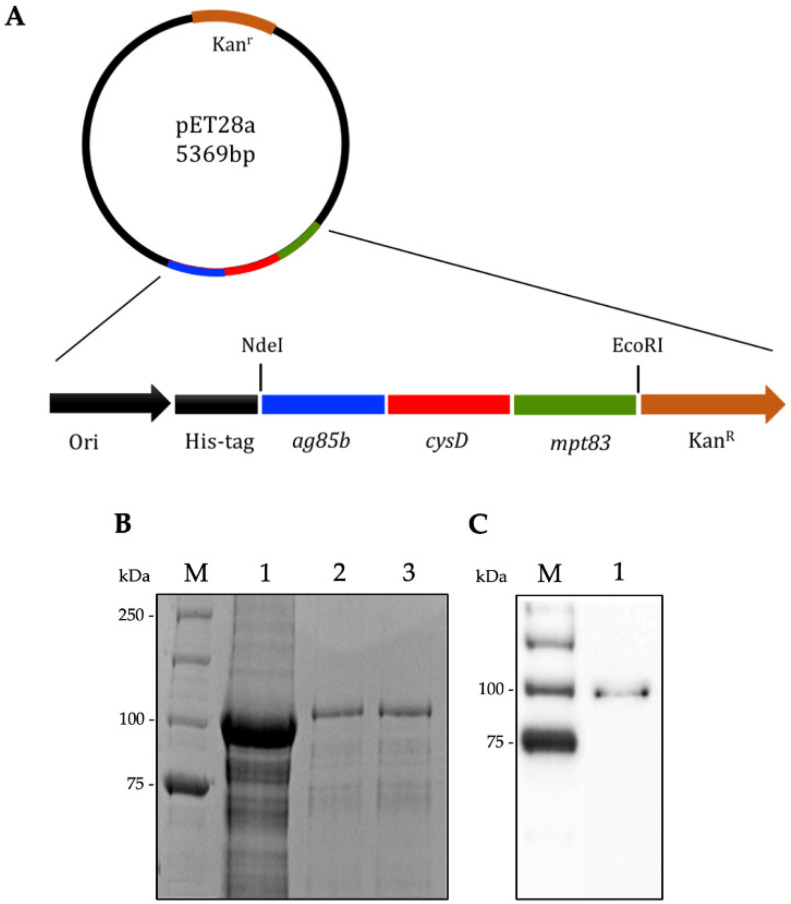
**Expression, purification and analysis of the CysVac5 recombinant protein.** (**A**) Structural diagram of the recombinant prokaryotic expression plasmid pET28a-CysVac5. (**B**) Purification of CysVac5 (molecular weight of 93 kDa) via Coomassie Blue-stained SDS-PAGE. *E. coli* BL21 expressing CysVac5 whole cell lysate (lane 1), purified and refolded CysVac5 (lane 2, 3), molecular weight ladder (M). (**C**) Purified CysVac5 was verified by immunoblot with His-tag monoclonal antibody–HRP conjugate (lane 1), molecular weight ladder (M).

Previous studies described the vaccine based on CysVac2 fusion protein, consisting of a fusion of Ag85B and CysD antigens [14]. We sought to improve on CysVac2 by adding an additional antigen, i.e., MPT83, making a tri-antigen fusion protein. The recombinant product, which was termed CysVac5 (Figure 1A), was expressed as the fusion protein with a molecular weight of approximately 100 kDa, purified from pET28a-CysVac5-transformed *E. coli* under denaturing conditions and refolded in a Tris-based buffer. SDS-PAGE (Figure 1B) and Western blot using a His-tag monoclonal antibody–HRP conjugate (Figure 1C) were used to verify the purity and specificity of the purified recombinant fusion protein.

**Figure 2:**
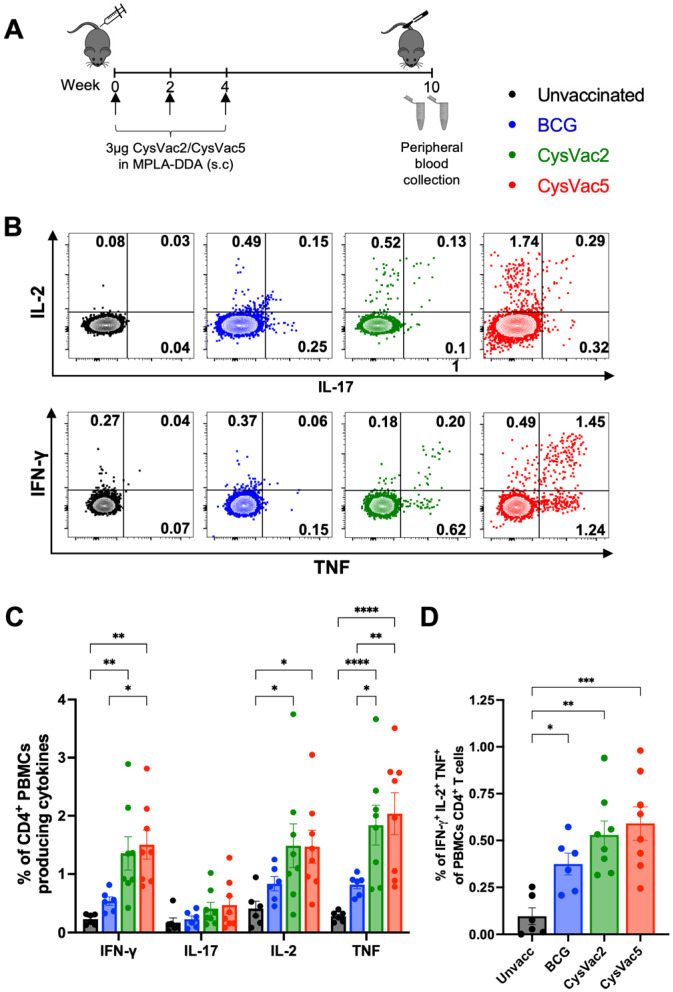
**Induction of cytokines producing CD4^+^ T cells in the PBMCs of C57BL/6 mice following CysVac5-MPLA/DDA vaccination.** (**A**) C57BL/6 mice (n = 6–8) were vaccinated once subcutaneously (s.c.) with 5 × 10^5^ CFU of BCG or 3 times s.c. with 3 µg of either CysVac2 or CysVac5 vaccines formulated in MPLA/DDA at 2-week intervals. Six weeks after the last vaccination, PBMCs were isolated, and intracellular cytokine staining was performed. Representative flow plots (**B**) demonstrating frequencies of IFN-γ, IL-17, IL-2 and/or TNF-positive CD4^+^ T cells (**C**,**D**). Data are representative of 2 independent experiments and show cytokine frequency ± SEM for each group. The significance of differences between the groups was determined by ANOVA with Tukey’s multiple comparisons test (* *p* < 0.1; ** *p* < 0.01; *** *p* < 0.001; **** *p* < 0.0001; see Supplementary Figure S1 for gating strategy).

In order to assess the immunogenicity of CysVac5, we vaccinated C57BL/6 mice subcutaneously with a fusion protein, either CysVac2 or CysVac5 formulated in the adjuvant combination of MPLA and DDA, as detailed in Figure 2A. The MPLA/DDA adjuvant was chosen because it induces strong protective responses, characterised by the Th1 and Th17 CD4^+^ T-cell response [15]. Six weeks after the last vaccination, peripheral blood mononuclear cells (PBMCs) were collected and restimulated ex vivo with CysVac5. The cytokine production was examined by intracellular staining followed by acquisition by flow cytometry. While we did not detect CD8^+^ T-cell responses above background, the CD4^+^ T-cell population in PBMCs of CysVac2 or CysVac5 vaccinated mice were able to secrete IFN-γ, IL-2 and TNF at significantly higher levels than in the unvaccinated group (Figure 2B,C). IL-17 in vaccinated groups was not significantly increased compared to unvaccinated mice (Figure 2C). The proportion of CysVac5-reactive CD4^+^ T-cells secreting multiple cytokines was also greater for the CysVac2 and CysVac5 vaccinated mice in comparison to the unvaccinated group (Figure 2D). This multifunctional population has been shown to be more protective in several pre-clinical studies [21].

### 3.2. CysVac5-MPLA/DDA Reduced the Bacterial Burden and Pathology in M. tuberculosis-Infected Mice

**Figure 3:**
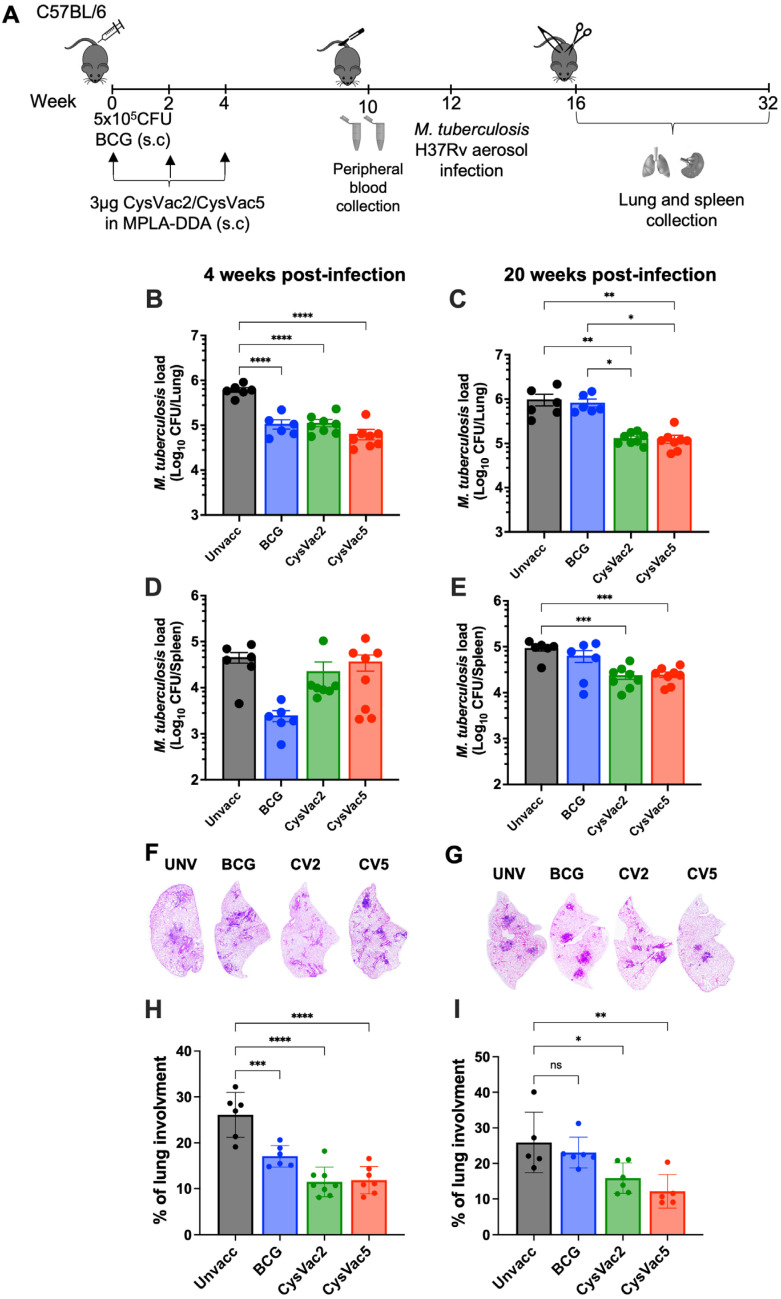
**Protection afforded by CysVac5 following aerosol *M. tuberculosis* infection.** C57BL/6 (n = 6–8) were vaccinated as shown, and 6 weeks after the last vaccination, mice were challenged with a low-dose aerosol of *M. tuberculosis* H37Rv (100 CFU/mice) (**A**). At 4 (**B**,**D**) or 20 (**C**,**F**) weeks post-infection, the bacterial loads were enumerated in the lungs (**B**,**C**) or spleen (**D**,**E**). Determination of pulmonary pathology using H&E (hematoxylin-eosin) visualisation and microscopy (**F**,**G**). Area of the lung is shown as a percentage occupied by total granulomatous tissue in the lung section of *M. tuberculosis*-infected mice (**H**,**I**). Data are the means ± SEM and are representative of 2 independent experiments. CV2 = CysVac2, CV5 = CysVac5. Statistical significance was determined by ANOVA with Tukey’s multiple comparisons test (* *p* < 0.1; ** *p* < 0.01; *** *p* < 0.001; **** *p* < 0.0001).

To determine if CysVac5 vaccination was protective, six weeks after the last vaccination, C57BL/6 mice were challenged with a low-dose aerosol of *M. tuberculosis* H37Rv (approximately 100 CFU per mouse). At 4 and 20 weeks after the infection, bacterial burden and lung pathology were assessed in the spleen and lungs of mice (Figure 3A). In the lungs, CysVac5 conferred a significant level of protection against *M. tuberculosis* infection at the early time point (4 weeks post-challenge) compared to the unvaccinated group with a 0.9 Log_10_ CFU reduction, similar to that observed after vaccination with BCG or CysVac2 (Figure 3B). BCG-induced immunity to control *M. tuberculosis* infection at extended time points waned over time; however, the protection provided by CysVac5 was maintained (20 weeks post-challenge; Figure 3C). In the spleen, there were no significant differences in protection observed across the vaccinated groups at 4 weeks post-*M. tuberculosis* infection (Figure 3D). However, at 20 weeks post-challenge, CysVac2- and CysVac5-immunised mice had significantly lower numbers of bacterial CFU compared to unvaccinated or BCG-immunised mice (Figure 3E).

At 4 weeks post-infection, the lung section of CysVac5-vaccinated mice appeared less inflamed, with darker and smaller lesions indicating more T-cell infiltration to the sites of infection and better bacterial containment (Figure 3F). Lung sections of CysVac5-vaccinated mice for the extended time point showed even cleaner areas compared to those collected from mice that were left unvaccinated or received only BCG vaccination (Figure 3G). CysVac2- and CysVac5-vaccinated mice had lower levels of granulomatous area at both early and extended time points after *M. tuberculosis* infection (Figure 3H,I), suggestive of less severe pathology when compared to mice that were not vaccinated or mice that received BCG. Overall, CysVac5 vaccination was protective against *M. tuberculosis* infection at both short (4 weeks) and extended (20 weeks) time points after challenge, with reduced lung pathology.

### 3.3. CysVac5-MPLA/DDA Vaccination Increases the Number of Antigen-Specific Cytokine-Secreting Cells Following M. tuberculosis Infection

**Figure 4:**
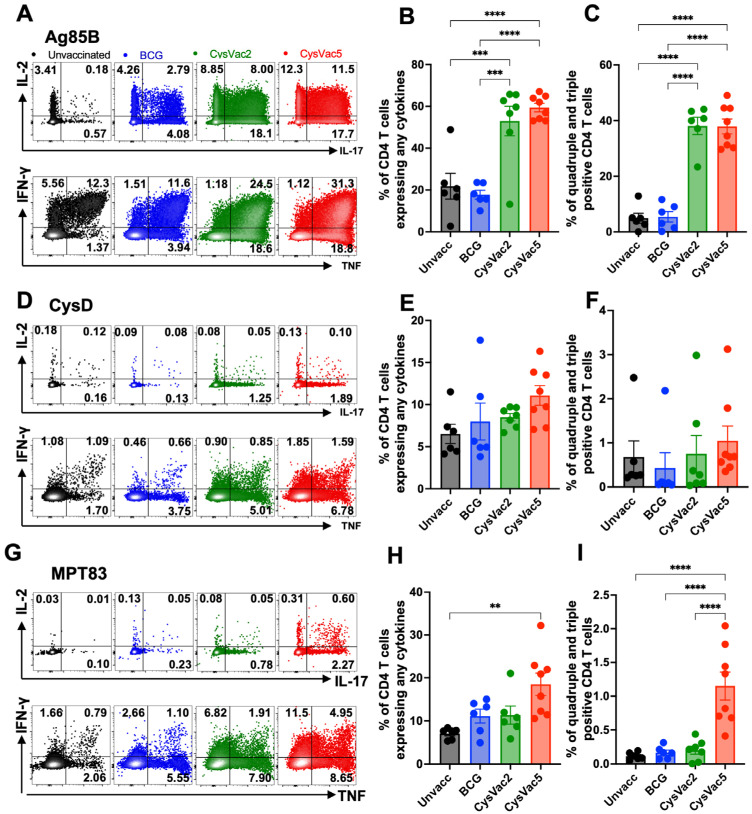
**Component-specific CD4^+^ T-cell responses in CysVac2-vaccinated mice.** C57BL/6 (n = 6–8) were vaccinated as per Figure 2. Six weeks after the last vaccination, mice were infected with a low-dose aerosol of *M. tuberculosis* H37Rv (100 CFU/mice). Four weeks post-infection, lung cells were restimulated with Ag85B (**A**–**C**), CysD (**D**–**F**) and MPT83 (**G**–**I**) in the presence of Brefeldin A and stained for surface markers and intracellular cytokines. Shown are representative dot plots from each group (**A**,**D**,**G**) and bar graphs for the percentage of CD4^+^ T cells expressing any cytokine (**B**,**E**,**H**) or expressing three or four cytokines simultaneously (**C**,**F**,**I**). Data are the means ± SEM and are representative of 2 independent experiments. Statistical significance was determined by ANOVA with Tukey’s multiple comparisons test (** *p* < 0.01; *** *p* < 0.001; **** *p* < 0.0001).

**Figure 5:**
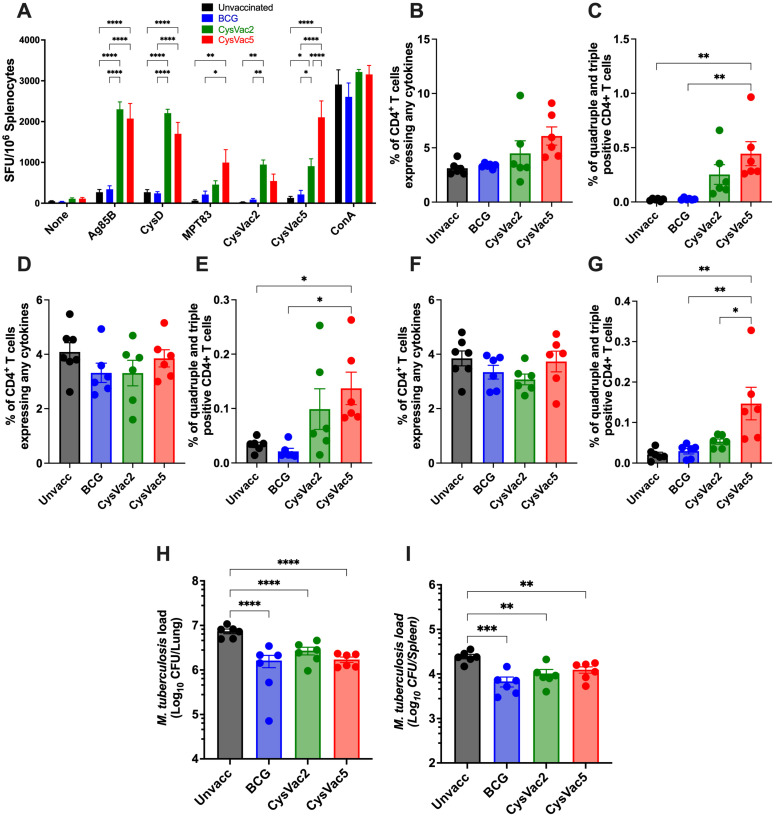
**Antigen-specific immune response and protection generated by CysVac5 in BALB/c mice.** BALB/c mice (n = 6) were vaccinated and infected with *M. tuberculosis,* as per Figure 3. Four weeks post-infection, ELISPOT was performed on splenocytes after recall to Ag85B, CysD, MPT83, CysVac2, CysVac5 or ConA (**A**). Lung cells were restimulated with Ag85B (**B**,**C**), CysD (**D**,**E**) or MPT83 (**F**,**G**) in the presence of Brefeldin A and stained for surface markers and intracellular cytokines. Shown are the percentage of CD4^+^ T cells expressing any cytokine (**B**,**D**,**F**) or expressing three or four cytokines simultaneously (**C**,**E**,**G**). The bacterial loads were enumerated at 4 weeks post-challenge in the lungs (**H**) and spleen (**I**). Data are the means ± SEM and are representative of 2 independent experiments. Statistical significance was determined by ANOVA with Tukey’s multiple comparisons test (* *p* < 0.1; ** *p* < 0.01; *** *p* < 0.001; **** *p* < 0.0001).

To characterise immunity generated by CysVac5 vaccination after *M. tuberculosis* infection, pulmonary cells were collected and restimulated ex vivo with each single-protein component of the CysVac5 fusion protein, i.e., either Ag85B or CysD or MPT83. Upon restimulation with Ag85B (Figure 4A), approximately 60% of the total CD4^+^ T cells were able to respond by producing any cytokine (Figure 4B), and approximately 40% of the total CD4^+^ T cells in the lungs were quadruple- or triple-positive for IFN-γ, IL-2, IL-17 and TNF (Figure 4C). This number was significantly higher compared to mice that received only BCG or were left unvaccinated. A similar trend was maintained when these subsets were observed after being stimulated ex vivo with the other vaccine component CysD (Figure 4D–F), although population sizes were smaller than those seen upon recalling to Ag85B. For MPT83 restimulation (Figure 4G–I), CysVac5-vaccinated mice were the only mice with significantly higher percentages of cytokine-secreting CD4^+^ T cells (Figure 5H). At 20 weeks after *M. tuberculosis* infection, mice that received CysVac5 had a higher frequency of CD4^+^ T cells producing any cytokines as well as a higher frequency of quadruple- and triple-positive CD4^+^ T cells compared to the unvaccinated group and the BCG-vaccinated group, and similar frequency to mice vaccinated with CysVac2 after recall to the Ag85B component (Supplementary Figure S2A). The specific response against CysD did not reveal any differences between groups at this time point despite having increased in frequency compared to the 4-week time point (Supplementary Figure S2B). The MPT83-specific CD4^+^ T cells in the lungs of immunised mice reflected the same patterns of responses seen at 4 weeks after the infection, with the CysVac5-vaccinated group being the only one with statistically significantly higher numbers of CD4^+^ T cells producing any cytokines (Supplementary Figure S2C). Together, these results indicate that CysVac5 vaccination formulated in MPLA/DDA adjuvant was able to stimulate both Th1 and Th17 responses against the three antigenic components of the fusion protein.

### 3.4. CysVac5-MPLA/DDA Induces Protective Immunity across Different MHC Haplotypes

Effective vaccines should be able to impart protection against infection/disease in populations with different genetic backgrounds. To examine this, BALB/c mice (I-A^d^/-I-E^d^) were either left unvaccinated or vaccinated, as in Figure 3A. Six weeks after the last vaccination, mice were infected with a low-dose aerosol of *M. tuberculosis* H37Rv; 4 weeks later, the bacterial load and antigen-specific immune responses were assessed in the lung and spleen.

Significantly higher numbers of antigen-specific IFN-γ splenic secreting cells were observed in the spleen for each of the single-component and the whole fusion proteins of the vaccine of CysVac2 and CysVac5 immunised mice (Figure 5A). In BALB/c mice, Ag85B was not immunodominant, with a similar magnitude of responses observed between Ag85B and CysD (Figure 5A). There was a significantly increased number of MPT83-specific cells producing IFN-γ cytokine found in the splenocytes of mice vaccinated with CysVac5 compared to the other three groups (Figure 5A). In the lung, cytokine responses against all three components of the fusion protein were comparable in magnitude. While the overall proportion of cytokine-secreting cells was similar for all groups irrespective of the recall antigen (Figure 5B,D,F), mice immunised with CysVac5 showed higher frequencies of quadruple- and triple-positive CD4^+^ T cells compared to those that received BCG or were left unvaccinated for all three antigens (Figure 5C,E,G). Quadruple- and triple-positive CD4^+^ T cells after recall to MPT83 were greater in CysVac5-vaccinated mice compared to those from CysVac2-vaccinated mice, reflecting the addition of this antigen to CysVac5 (Figure 5G).

In BALB/c mice, CysVac5 was able to reduce the bacterial burden in both lungs (Figure 5H) and spleen (Figure 5I) compared to the unvaccinated mice. No difference was observed between CysVac5-vaccinated and BCG-vaccinated mice. Likewise, similar protection was observed after vaccination with CysVac5 or CysVac2. Overall, these results demonstrate that CysVac5 could generate a broader immune response than CysVac2 against *M. tuberculosis* infection across multiple MHC haplotypes.

## 4. Discussion

The selection of novel and optimal antigens for inclusion in subunit vaccines is a critical step in TB vaccine design and development. However, the choice of antigens used in *M. tuberculosis* vaccine candidates that are now in clinical trials has been primarily focusing on a small subset of immunodominant proteins, such as antigen 85 complex, ESAT6 or TB10.4 [22,23]. In this study, we characterised the CD4^+^ T cell immunogenicity and the protective efficacy of a fusion protein consisting of three different protein components, namely Ag85B, CysD and MPT83. The fusion protein, termed CysVac5, was based on CysVac2, the first generation of the CysVac multistage vaccine family whose components are expressed at different stages of the *M. tuberculosis* life cycle and provided significant protection against *M. tuberculosis* infection in several murine models [14,15,24]. In this study, the antigen MPT83 was included in the CysVac2 recombinant protein to form a novel fusion protein termed CysVac5. MPT83 (Rv2873) is a secreted mycobacterial lipoprotein expressed on the surface of *M. tuberculosis*. Although its function is not yet well characterised, this antigen is recognised by T cells after *M. tuberculosis* infection in both mice and humans [18]. When mice were vaccinated with either protein or DNA vaccine, MPT83 conferred significant levels of protection in both lungs and spleen against aerosol *M. tuberculosis* infection, with strong IFN-γ CD4^+^ and CD8^+^ T-cell responses [18]. With the addition of another immunogenic antigen, MPT83, to the fusion vaccine, it was hypothesised that CysVac5 could provide an improved immune response and protective efficacy against TB infection compared to CysVac2.

The formulation of CysVac5 in a specific liposome construction MPLA/DDA adjuvant resulted in a vaccine that induced polyfunctional CD4^+^ T cells secreting multiple cytokines in mice both pre- and post-*M. tuberculosis* challenge and a reduced bacterial number in the lungs and spleen. Subcutaneous injection of CysVac5 formulated in MPLA/DDA adjuvant in C57BL/6 mice resulted in the generation of a mix of Th1 and Th17 responses both before and after *M. tuberculosis* infection, indicated by high frequencies of CD4^+^ T cells producing multiple cytokines (IFN-γ, IL-2, IL-17 and TNF). The induction of T cells producing IFN-γ, IL-2 and TNF (Th1 response) observed in CysVac5-vaccinated mice (Figure 2) correlates with vaccine-induced Th1-mediated protection, as numerous studies have demonstrated the correlation of these responses with protective immunity in animal models, and evidence is accumulating on the important contribution of IL-17 to anti-TB immunity [15,25,26]. In humans, it has been shown that stronger mycobacteria-specific polyfunctional CD4^+^ T cells were found in adults with smear-negative sputum for acid-fast bacilli (AFB) than those with AFB smear-positive TB. In addition, successful TB treatments that rapidly reduce the bacterial load are associated with an increased proportion of this polyfunctional subset. The increased population of PPD-specific CD4^+^ T cells was also reported to correlate with the significant inhibition of mycobacterial growth in BCG-vaccinated infants [27]. On the other hand, several studies have demonstrated that polyfunctional CD4^+^ did not correlate with protection and suggested that other functional attributes, such as tissue homing potential and additional effector functions, may play a bigger role in promoting protection [28]. Thus, further study is required to define the true role of polyfunctional CD4^+^ T cells in anti-TB immunity. Further, we did not measure the induction of vaccine-specific antibodies in this study, which may play an accessory role in protection against *M. tuberculosis* infection [29].

Ag85B is a secreted immunogenic antigen that has been used widely in developing subunit protein vaccines for TB. This antigen contains numerous well-characterised epitopes recognised in *M. tuberculosis* infection models, as well as in TB patients [30]. Several studies have shown that Ag85B displays the most robust antigenicity, with an epitope that binds to 13 major histocompatibility complexes [31]. This might explain the high percentage of polyfunctional CD4^+^ T cells observed in both CysVac5- and CysVac2-vaccinated mice following stimulation with Ag85B protein antigen (Figure 3). Following recall to CysD or MPT83, the immune responses were not as strong as those seen when stimulated with Ag85B. Various factors are to be considered as to why CysD/MPT83 appeared to be less immunogenic, such as how major epitopes/antigenic determinants of these antigens relate to the issue of antigenic competition, optimal antigen presentation and the cellular environment for facilitating antigen-specific T cells [32]. There are also linked effects of host genetic background and mycobacterial pathogen on infection susceptibility [33]. Several epidemiological studies have shown that genetic factors have significant contributions to TB disease in humans. Multiple series and extensive experimental studies in various animal models have also established the importance of host genetic background in assessing the outcomes of *M. tuberculosis* infection [34]. Indeed, in this study, CysVac5 vaccination could provide protection against TB infection in mice with different genetic backgrounds. Similar findings were observed in a previous study determining the capacity of CysVac2 in inducing protection against aerosol *M. tuberculosis* infection in C57BL/6, BALB/c and outbred mice [14]. In BALB/c mice, CysVac5 was able to elicit strong mixed Th1/Th17 responses both before and after challenge. This was shown by the increased populations of multifunctional CD4^+^ T cells producing IFN-γ, IL-2 and TNF, as well as IL-17, in the PBMCs or lungs of vaccinated mice (Figure 5). This immunity correlated with protection against *M. tuberculosis* infection in both the lungs and the spleens of mice *(*Figure 5).

## 5. Conclusions

Overall, this study demonstrates that vaccination with CysVac5 significantly enhances the protective efficacy against *M. tuberculosis* challenge in mice. This protection efficacy is evident at early time points, as well as at extended time points after *M. tuberculosis* infection, when the protection of BCG started waning. Furthermore, the protective immunity, characterised by a mix of Th1 and Th17, is maintained across multiple murine strains. A new TB vaccine candidate should also demonstrate consistent, robust and high-level efficacy across a range of animal models before it advances to clinical trials, warranting the assessment of CysVac5 in additional models, such as the guinea pig and/or the NHP [35].

## Data Availability

The data that support the findings of this study are available from the corresponding author upon reasonable request.

## Supplementary Materials

The following supporting information can be downloaded at: https://www.mdpi.com/article/10.3390/vaccines12090997/s1. Table S1: Monoclonal antibodies used for CD4^+^ T cell analysis. Table S2: Monoclonal antibodies used to assess antigen-specific cytokine production. Figure S1: Gating strategy used for assessing intracellular cytokine release by T cells. Figure S2: Component-specific CD4^+^ T cell responses 20 weeks post-infection. Figure S3: Cytokine responses after CysVac2 vaccination. Figure S4: Immunogenicity of CysVac5 in BALB/c mice. Figure S5: Enlarged H&E (hematoxylin-eosin) images from Figure 3.
