# Supplementary material for: Immunogenicity and Protective Efficacy of a Multi-Antigen Mycobacterium tuberculosis Subunit Vaccine in Mice
Source: Vaccines (Basel). 2024 Aug 30;12(9):997. doi: 10.3390/vaccines12090997 (PMC11435920; doi:10.3390/vaccines12090997)
Supplement: Supplementary file 1 [file vaccines-12-00997-s001.zip › vaccines-3124517-supplementary.pdf]

Supporting Information

Immunogenicity and protective efficacy of a multi-antigen *Mycobacterium tuberculosis* subunit vaccine in mice

Nisa *et. al.*

Supplementary Table S1. Monoclonal antibodies used for CD4⁺ T cell analysis.

Marker	Clone	Fluorochrome	Dilution	Manufacturer
B220	PA3-6B2	PerCP/Cy5.5	1 in 200	BD Pharmingen
CD4	RM4-5	AF700	1 in 200	BD Pharmingen
CD44	IM7	PE/Cy7	1 in 300	BD Pharmingen
CD62L	MEL-14	PE	1 in 300	BioLegend
CD8a	53-6.7	APC-Cy7	1 in 200	BD Pharmingen
FcRIII/II	2.4G2	NA	1 in 200	BD Pharmingen
KLRG-1	2F1/KLRG1	FITC	1 in 300	BioLegend
RorγT	Q31-378	PECF594	1 in 200	BD Horizon
Tbet	4B10	APC	1 in 200	BioLegend

Supplementary Table S2. Monoclonal antibodies used to assess antigen-specific cytokine production.

Marker	Clone	Fluorochrome	Dilution	Manufacturer
CD4	RM4-5	AF700	1 in 200	BD Pharmingen
CD44	IM7	FITC	1 in 300	BD Pharmingen
CD8a	53-6.7	APC-Cy7	1 in 200	BD Pharmingen
FcRIII/II	2.4G2	NA	1 in 200	BD Pharmingen
IFN-γ	XMG1.2	PE/Cy7	1 in 300	BD Pharmingen
IL-17	TC11-18H10	Pacific Blue	1 in 200	BD Horizon
IL-2	JES6-5H4	PE	1 in 200	Miltenyi Biotec
TNF	MP6-XT22	PerCP/Cy5.5	1 in 200	BD Pharmingen

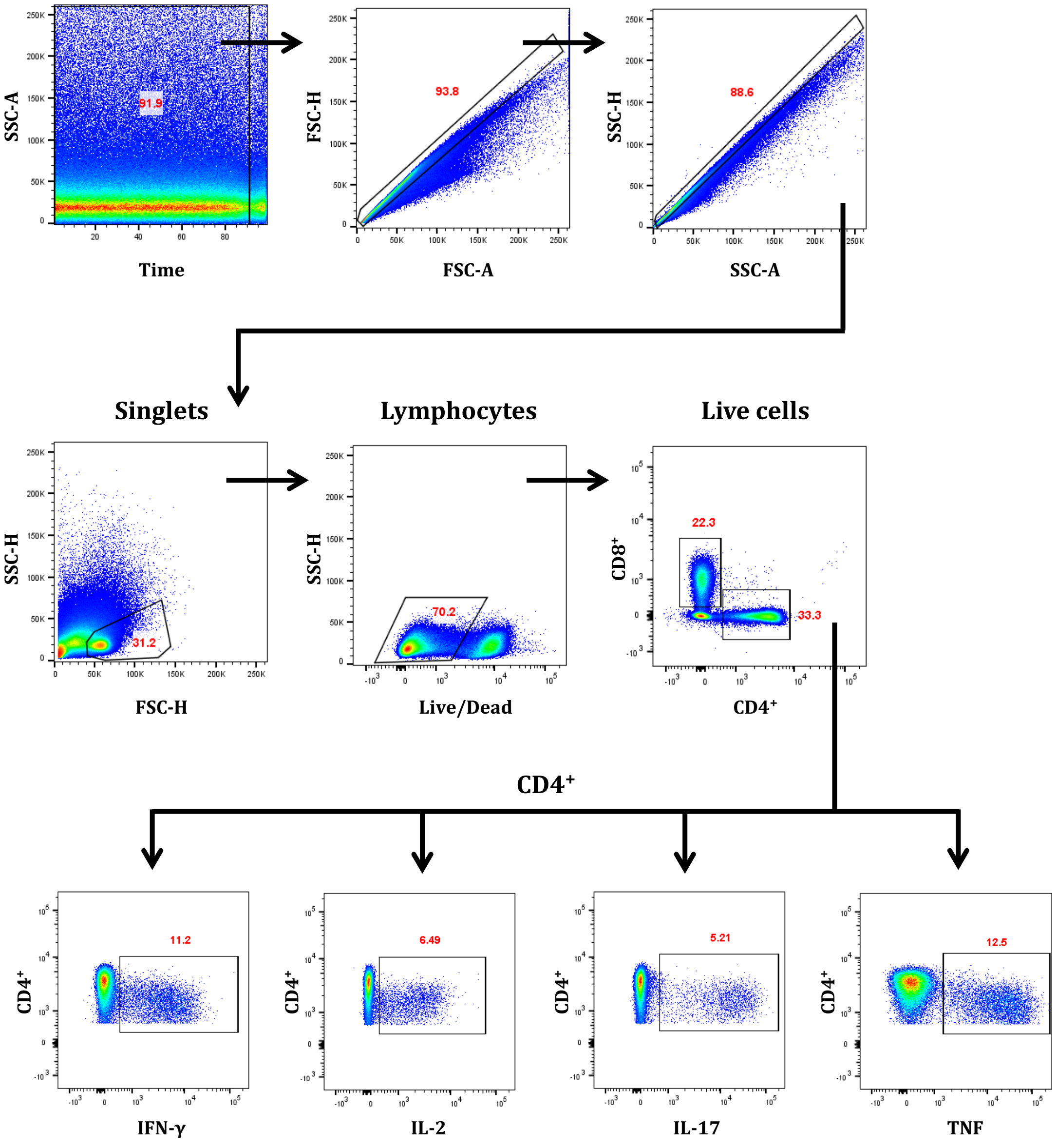

Supplementary Figure S1. Gating strategy used for assessing intracellular cytokine release by T cells. A time gate was applied to verify consistent fluorescence signal during sample acquisition. After excluding debris by FSC and SSC, dead cells were excluded by live/dead UV stain. The $CD4^{+}$ and $CD8^{+}$ T cell populations were gated, and the expression of IFN-γ, IL-2, IL-17 or TNF was gated for CD4+ T cells. To quantitate cells producing multiple cytokines, a Boolean analysis was applied.

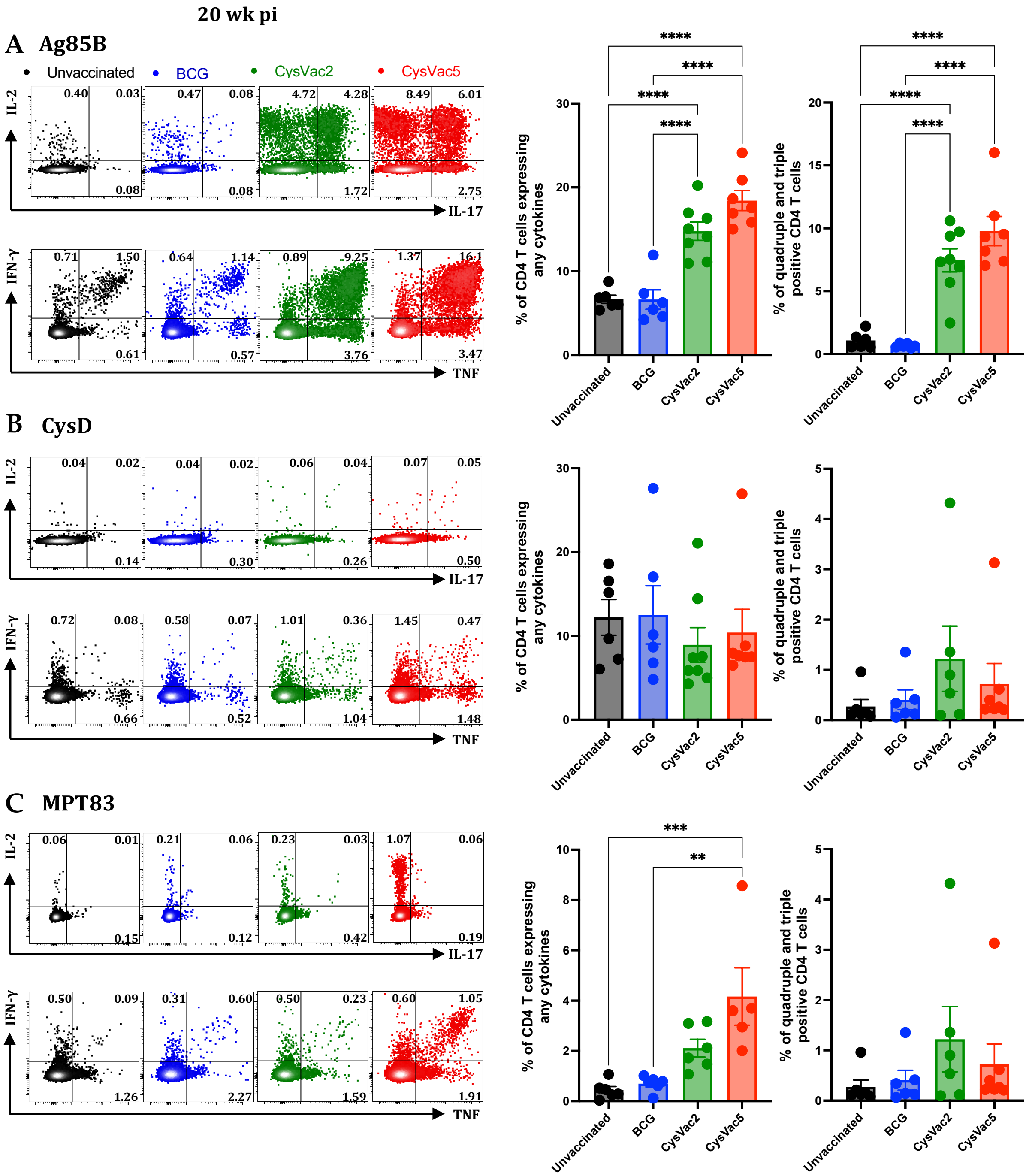

Supplementary Figure S2. Component-specific CD4+ T cell responses 20 weeks post-infection. C57BL/6 mice (n=6-8) were vaccinated as per Figure 2A. Six weeks after last vaccination mice were infected with a low dose aerosol of *M. tuberculosis* H37Rv (100 CFU/mice). Four weeks post-infection, lung cells were restimulated with Ag85B (A), CysD (B), and MPT83 (C) in the presence of Brefeldin A, and stained for surface markers and intracellular cytokines. Each panel shows a representative dot plot from each group, and a bar graph for the percentage of CD4+ T cells expressing any cytokine or expressing three or four cytokines at once. Data are the means ± SEM and are representative of 2 independent experiments. Statistical significance was determined by ANOVA with Tukey's multiple comparisons test (* p<0.1; ** p<0.01; *** p<0.001; **** p<0.0001).

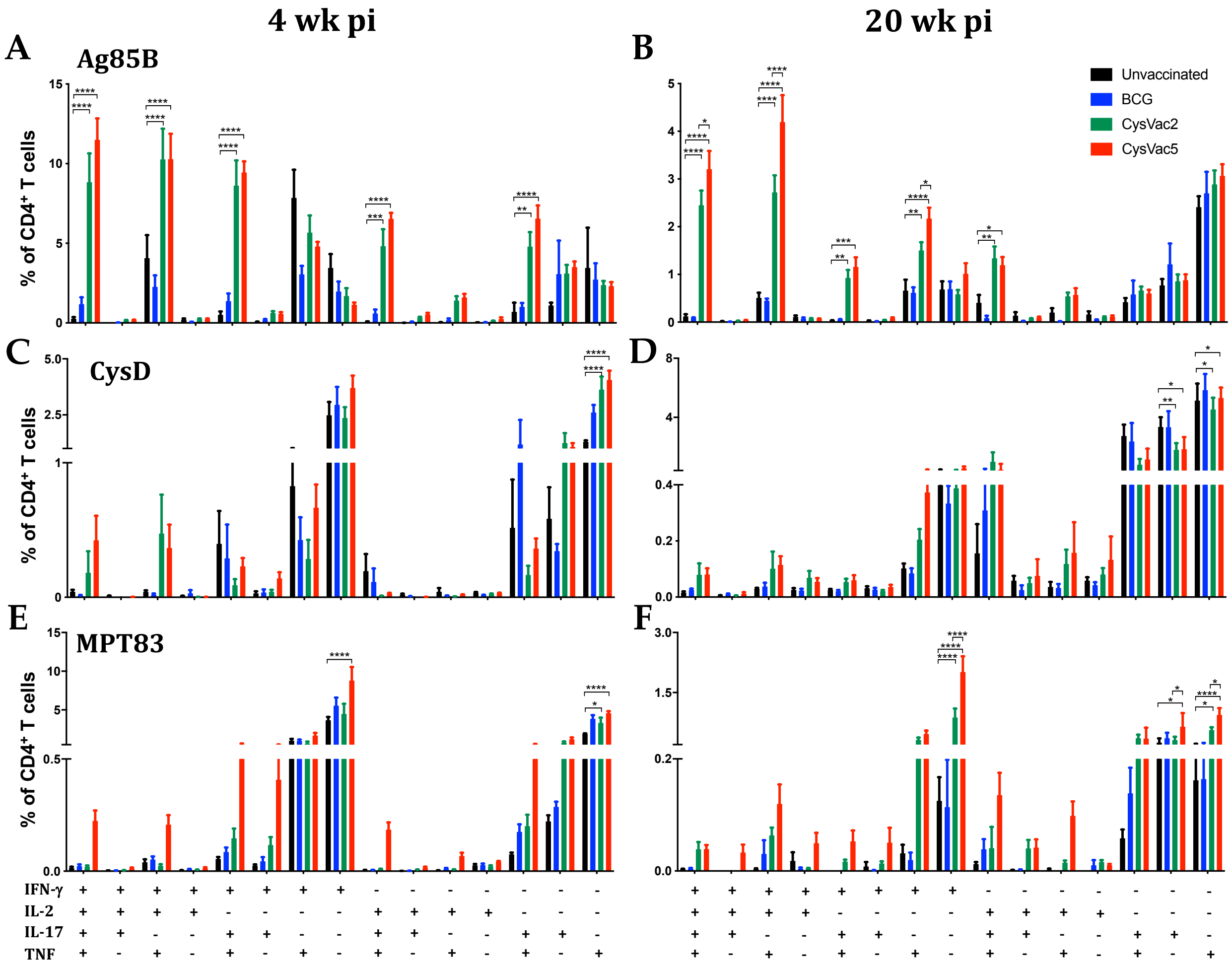

Supplementary Figure S3. Cytokine responses after CysVac2 vaccination. C57BL/6 mice (n=6-8) were vaccinated as per Figure 2A. Six weeks after last vaccination mice were infected with a low dose aerosol of M. tuberculosis H37Rv (100 CFU/mice). Four (A, C, E) and 20 weeks post-infection (B, D, F), lung cells were restimulated with Ag85B (A,B), CysD (C,D), and MPT83 (E,F) in the presence of Brefeldin A, and stained for surface markers and intracellular cytokines. Percentages of subpopulation of CD4+ T cells producing different combinations of cytokines (INF-g, IL-2, IL-17 or TNF) were calculated by Boolean gating using FlowJo. Data are the means ± SEM and are representative of 2 independent experiments. Statistical significance was determined by ANOVA with Tukey's multiple comparisons test (* p<0.1; ** p<0.01; *** p<0.001; **** p<0.0001).

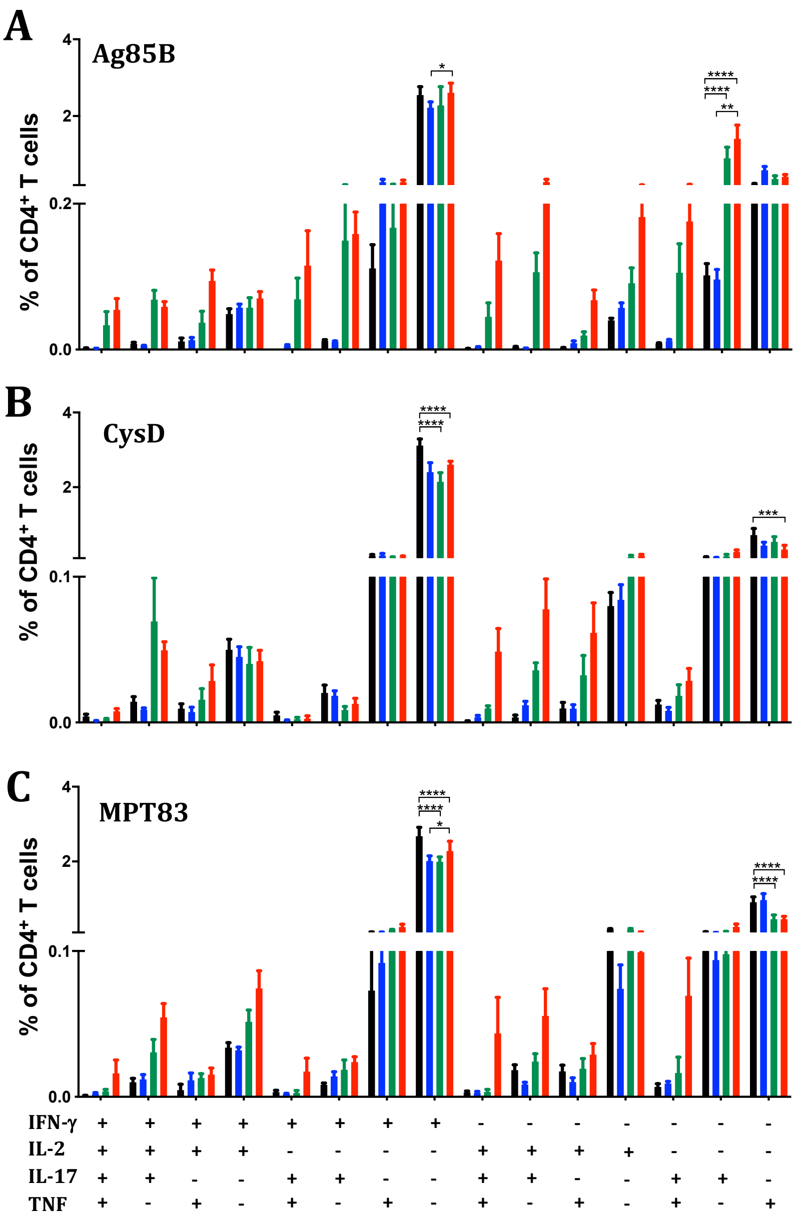

Supplementary Figure S4. Immunogenicity of CysVac5 in BALB/c mice. BALB/c mice (n=6-8) were vaccinated as per Figure 2A. Six weeks after last vaccination mice were infected with a low dose aerosol of *M. tuberculosis* H37Rv (100 CFU/mice). Four weeks post-infection, lung cells were restimulated with Ag85B (A), CysD (B), and MPT83 (C) in the presence of Brefeldin A, and stained for surface markers and intracellular cytokines. Percentages of subpopulation of CD4+ T cells producing different combinations of cytokines (INF-g, IL-2, IL-17 and TNF) were calculated by Boolean gating using FlowJo. Data are the means ± SEM and are representative of 2 independent experiments. Statistical significance was determined by ANOVA with Tukey's multiple comparisons test (* p<0.1; ** p<0.01; *** p<0.001; **** p<0.0001).

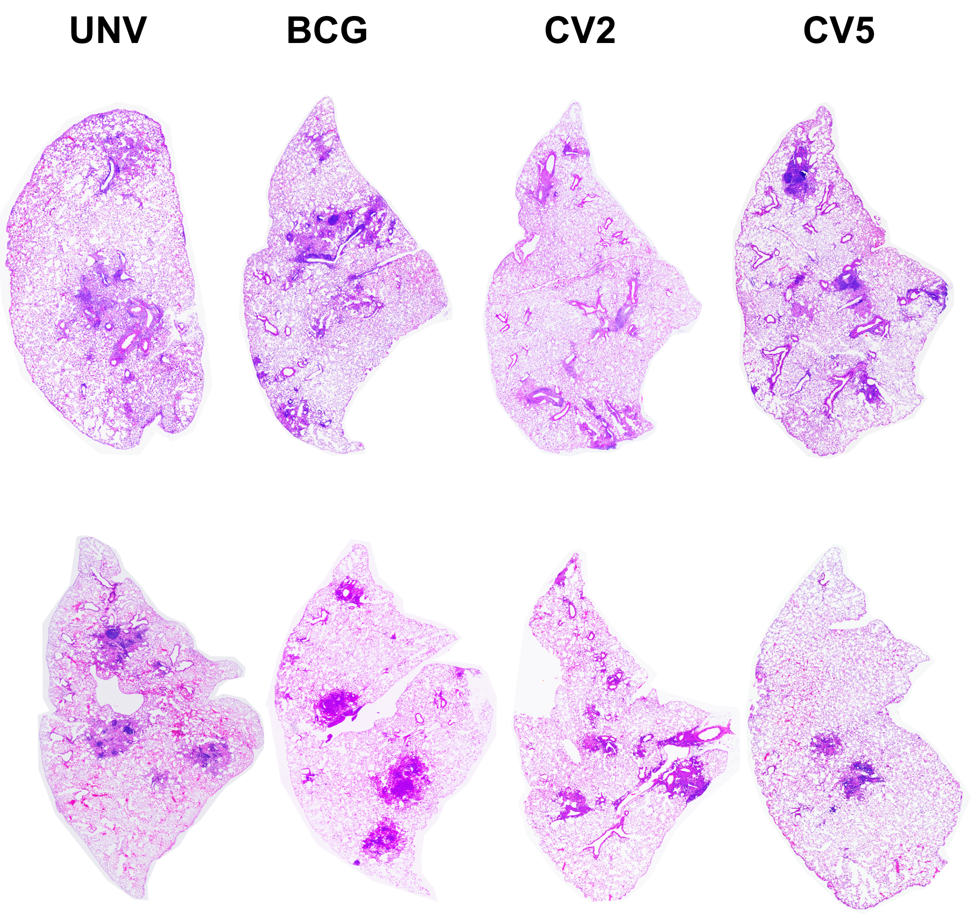

Supplementary Figure S5. Enlarged H&E (hematoxylin-eosin) images from Figure 3.
